# Amino Sugar-Enriched Fraction of Korean Red Ginseng Extract Induces the Priming Step of NLRP3 Inflammasome

**DOI:** 10.3390/molecules29071455

**Published:** 2024-03-24

**Authors:** Huijeong Ahn, Geun-Shik Lee

**Affiliations:** College of Veterinary Medicine and Institute of Veterinary Science, Kangwon National University, Chuncheon 24341, Republic of Korea; balloon1981@naver.com

**Keywords:** amino sugars, NLRP3, interleukin 1β, Korean Red Ginseng

## Abstract

Intracellular protein complexes, known as inflammasomes, activate caspase-1 and induce the secretion of pro-inflammatory cytokines, namely interleukin (IL)-1β and -18. Korean Red Ginseng extract (RGE) is a known immunomodulator and a potential candidate for the regulation of inflammasomes. The saponins, such as ginsenosides, of RGE inhibit inflammasome signaling, while non-saponin substances containing amino sugars promote the priming step, up-regulating inflammasome components (pro-IL-1β, NLRP3, caspase-1, and Asc). In this study, the amino sugar-enriched fraction (ASEF), which increases only non-saponin components, including amino sugars, without changing the concentration of saponin substances, was used to investigate whether saponin or non-saponin components of RGE would have a greater impact on the priming step. When murine macrophages were treated with ASEF, the gene expression of inflammatory cytokines (*IL-1α*, *TNFα*, *IL-6*, and *IL-10*) increased. Additionally, ASEF induced the priming step but did not affect the inflammasome activation step, such as the secretion of IL-1β, cleavage of caspase-1, and formation of Asc pyroptosome. Furthermore, the upregulation of gene expression of inflammasome components by ASEF was blocked by inhibitors of Toll-like receptor 4 signaling. Maltol, the main constituent of ASEF, promoted the priming step but inhibited the activation step of the inflammasome, while arginine, sugars, arginine–fructose–glucose, and fructose–arginine, the other main constituents of ASEF, had no effect on either step. Thus, certain amino sugars in RGE, excluding maltol, are believed to be the components that induce the priming step. The priming step that prepares the NLRP3 inflammasome for activation appears to be induced by amino sugars in RGE, thereby contributing to the immune-boosting effects of RGE.

## 1. Introduction

Inflammasomes are multi-protein assemblies expressed in the cells of the immune system and epithelial cells [1,2]. Inflammasomes trigger the activation of inflammatory caspases, such as caspase-1, which promote the secretion of pro-inflammatory cytokines (e.g., interleukin [IL]-1β and IL-18) in response to cytosolic danger signals [1]. Inflammasome activation is a two-step process of priming and activation. In the priming step, the Toll-like receptor (TLR), tumor necrosis factor α-receptor (TNFR), and IL-1 receptor (IL-1R) signaling pathways upregulate the transcription of the pro-form of the cytokines and sensor proteins (e.g., nucleotide-binding oligomerization domain [NOD], leucine-rich repeat [LRR], and pyrin domain-containing 3 [NLRP3]) through nuclear factor (NF)-κB and others [1,3]. On the other hand, the activation step induces the assembly of inflammasome components (e.g., NLRP3, apoptosis-associated speck-like protein containing a caspase recruitment domain [ASC], and caspase-1) in response to triggers such as nigericin and adenosine triphosphate (ATP). The activation of the NLRP3 inflammasome leads to the maturation and secretion of IL-1β and IL-18, and the induction of inflammatory cell death (i.e., pyroptosis) [2]. NLRP3 inflammasomes have been known to play a role in the pathogenesis of various infectious (e.g., COVID-2019) and non-infectious diseases (e.g., diabetes, arteriosclerosis, and Alzheimer’s diseases) and there has been significant progress in research on the regulators of inflammasome activation [1,3,4]. 

Ginseng (*Panax ginseng* Meyer) is a well-known herbal medicine with several health benefits, including immunity-boosting properties [5]. Korean Red Ginseng is made by steaming and drying the roots of fresh ginseng, which enhances its efficacy, safety, and preservation [5]. Korean Red Ginseng extract (RGE) contains various bioactive components, such as saponins, polysaccharides, peptides, fatty acids, mineral oils, and amino sugars, with pharmacological properties. Among these, research on the saponins of ginseng, ginsenosides, has been the most extensive [3,5]. Ginsenosides are well known for their anti-inflammatory effects that are exerted through the inhibition of pro-inflammatory cytokine expression. On the other hand, the other substances are known to enhance the phagocytic activity of macrophages against pathogens [5,6]. RGE has been reported to selectively inhibit NLRP3, absent in melanoma 2 (AIM2), and non-canonical (i.e., caspase-4/5 in humans and caspase-11 in mice) inflammasomes, and it is known that ginsenosides have this inhibitory effect [3,4]. 

In an earlier study, RGE was split into saponin and non-saponin fractions and their roles in inflammasome activation were tested [7]. The saponin fractions, containing high levels of ginsenosides, inhibited both the priming and activation steps. However, the non-saponin fractions of RGE, with significantly depleted levels of ginsenosides, induced the priming step for activation of the NLRP3 inflammasome [7]. Interestingly, the non-saponin fractions inhibited AIM2 inflammasomes, and maltol, a non-saponin substance, attenuated NLRP3 and caspase-11 inflammasomes [8,9]. However, the specific substance in the non-saponin fraction that promotes the priming step of inflammasome activation has not yet been determined. Therefore, this study investigated the effects of the amino sugar-enriched fraction (ASEF) of RGE on inflammasome activation to understand which component, saponin or non-saponin components of RGE, plays a more crucial role in regulating the priming step in NLRP3 inflammasomes. The ASEF contains increased amino sugars such as arginine–fructose–glucose (AFG), fructose–arginine (FA), and maltol, without significant changes in the total amount of ginsenosides. The ASEF treatment was carried out during the two distinct steps of NLRP3 inflammasome activation in mouse macrophages and the role of the ASEF on inflammasome activation and its mechanism were studied. The results of this study provide a scientific basis for the immune-boosting effects of RGE. 

## 2. Results

### 2.1. ASEF Increases the Expression of Cytokines and NLRP3 Inflammasome Components

**Figure 1:**
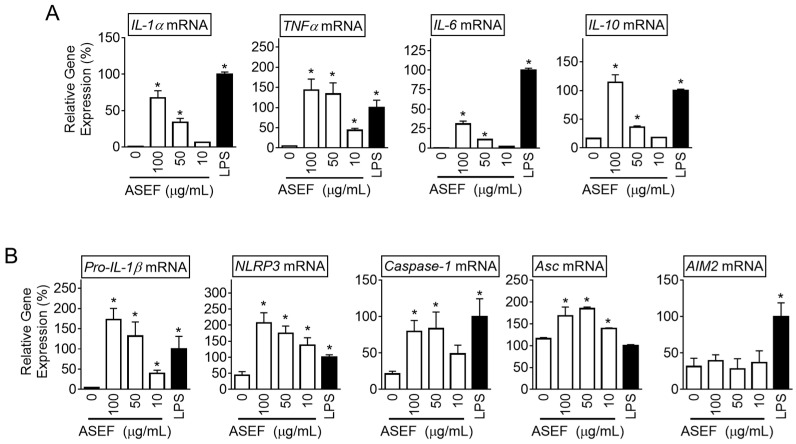
Effect of the ASEF on the gene expression of cytokines and inflammasome components. The BMDMs were treated with the indicated concentrations of the ASEF or LPS (10 ng/mL) for 3 h. (**A**): The gene expression levels measured by quantifying the mRNAs of *IL-1α*, *TNFα*, *IL-6*, and *IL-10* were analyzed by qPCR. (**B**): The levels of inflammasome components (i.e., *pro-IL-1β*, *NLRP3*, *caspase-1*, *Asc*, and *AIM2* genes) were assayed with qPCR. The bar graph presents the mean ± SD. All data shown are representative of at least three independent experiments. *, *p* < 0.05 vs. the non-treated group (0).

To study the effect of ASEF on the priming step of NLRP3 inflammasomes, the bone marrow-derived macrophages (BMDMs) from the mice were treated with the ASEF (10, 50, and 100 µg/mL) and the expression of cytokines was measured. The results showed that the ASEF dose-dependently upregulated the transcription of *IL-1α, TNF-α*, *IL-6*, and *IL-10* (Figure 1A and Supplementary Figure S1A). In addition, lipopolysaccharides (LPSs) as a positive control also induced the cytokine gene expression. LPS, a TLR4 ligand, is a well-known agent for the priming of inflammasome activation [10]. Based on this, it was evaluated whether the ASEF induced the priming of inflammasome activation. As seen in Figure 1B and Supplementary Figure S1A, the ASEF upregulated the expression of the *pro-IL-1β*, *NLRP3, caspase-1*, and *Asc* genes. However, the ASEF did not affect the expression of the *AIM2* gene, while LPS increased the *AIM2* transcripts. Taken together, the ASEF may be a trigger to induce cytokine expression and the priming step of the NLRP3 inflammasome. 

### 2.2. ASEF Induced the Levels of NLRP3, pro-IL-1β, and caspase-1

**Figure 2:**
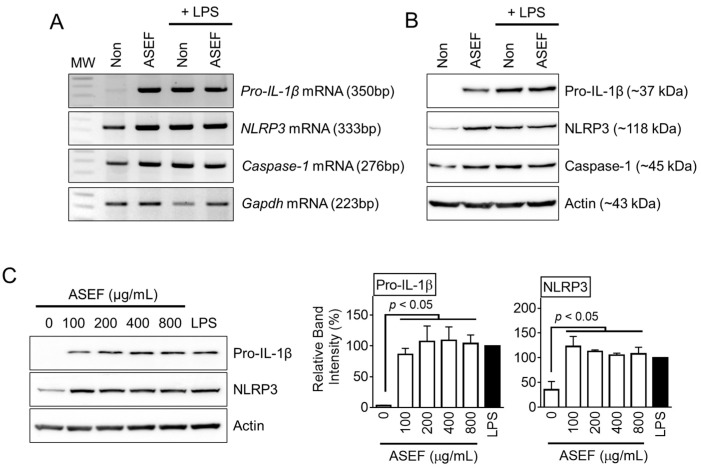
Effect of ASEF on the transcription and translation of inflammasome components. The BMDMs were treated with the ASEF (100 µg/mL) and/or LPS (10 ng/mL) for 3 h. The mRNA expression (**A**) was analyzed by RT-PCR and the protein levels (**B**) were measured through immunoblotting. (**C**) The BMDMs were treated with the indicated concentrations of the ASEF or LPS (10 ng/mL) for 3 h, after which the protein expression was analyzed by immunoblotting. The bar graphs present the band intensity. The bar graphs present the mean ± SD. All data shown are representative of at least three independent experiments.

It was further evaluated whether the ASEF and LPS present any synergistic or inhibitory effects on the gene expression of the NLRP3 inflammasome components. The BMDMs were treated with the ASEF and/or LPS, and the mRNA (Figure 2A) and protein (Figure 2B) expression of *pro-IL-1β*, *NLRP3*, and *caspase-1* were observed. The results showed that the ASEF and LPS individually induced similar levels of expressions of *pro-IL-1β*, *NLRP3*, and *caspase-1*. However, the ASEF and LPS together did not induce any additional upregulation of the three genes. Based on this, it could be inferred that the ASEF and LPS induced gene expression through the same pathways. Next, the effect of increasing the dosage of the ASEF on the upregulation of the NLRP3 inflammasome components was tested. The BMDMs were treated with increasing concentrations of the ASEF, and the levels of pro-IL-1β and NLRP3 proteins were detected. As shown in Figure 2C, the increasing dosages of the ASEF did not additionally upregulate the expression of the pro-IL-1β and NLRP3 proteins. The efficacy of the ASEF (100 µg/mL) on *pro-IL-1β* and *NLRP3* up-regulation was comparable to that of LPS. Taken together, these results show that the ASEF induces the priming step of NLRP3 inflammasome activation.

### 2.3. ASEF Stimulated the TLR4 Signaling Pathway, Resulting in the Upregulation of NLRP3 Inflammasome Components

**Figure 3:**
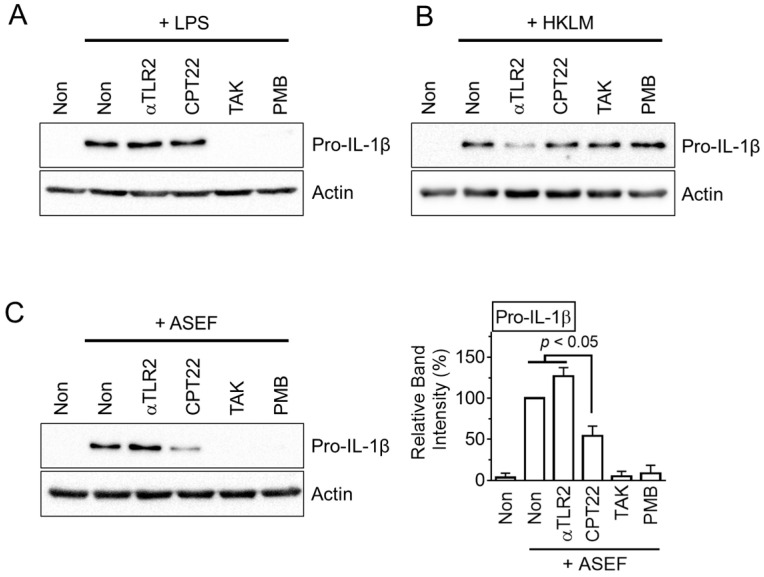
The study on the TLR signaling involved in ASEF-mediated priming step. The BMDMs were treated with LPS (**A**), HKLM (**B**), and the ASEF (**C**) in the presence of CU-CPT22 (CPT22), anti-TLR2 antibody (αTLR2), TAK, and PMB for 3 h. The protein levels of pro-IL-1β were measured with immunoblotting. The bar graph below presents the band intensity and the mean ± SD. All data shown are representative of at least three independent experiments.

The upregulation of the NLRP3 inflammasome components during the priming step occurred through several signaling pathways, including the TLR signaling pathway [11]. TLR inhibitors were adopted to test which TLR took part in the ASEF-mediated gene expression. As expected, a TLR4 ligand (i.e., LPS) induced the pro-IL-1β expression, which was blocked by the TLR4 inhibitor, TAK-242 (TAK), and the LPS inhibitor, polymyxin B (PMB), as shown in Figure 3A. In addition, the TLR2 ligand, heat-killed *Listeria monocytogenes* (HKLM), also upregulated pro-IL-1β proteins, and these were attenuated by the TLR2 inhibitor, anti-TLR2 antibody (Figure 3B). The ASEF treatment induced the expected level of pro-IL-1β expression. The ASEF-mediated pro-IL-1β expression was strongly inhibited by TAK and PMB, as well as partially decreased by the TLR1/2 inhibitor, CU-CPT22 (Figure 3C and Supplementary Figure S1B). Similarly, the ASEF-mediated NLRP3 expression was also inhibited by TAK, PMB, and CU-CPT22 (Supplementary Figure S1B). Through mechanistic studies, it was found that the ASEF mainly induces the priming step of the inflammasome through the TLR4 signaling pathway like LPS, with a low level of involvement of the TLR1 signaling pathway.

### 2.4. Maltol, an Amino Sugar in RGE, Induced the Expression of pro-IL-1β and NLRP3 Inflammasome Components

**Figure 4:**
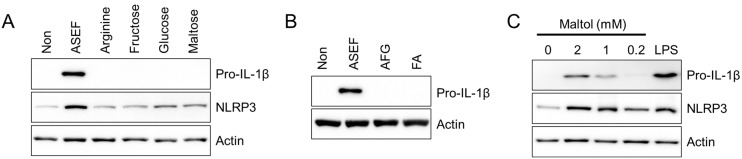
Effect of the arginine, sugars, and amino sugars on the priming step. (**A**,**B**): BMDMs were treated with arginine, fructose, glucose, maltose, AFG, and FA (each 1 mg/mL) for 3 h. (**C**): BMDM were treated with maltol as indicated for 3 h. The protein levels of pro-IL-1β and NLRP3 were measured with immunoblotting. All data shown are representative of at least three independent experiments.

Next, the effect of the major ingredients of the ASEF, such as arginine, sugars, and amino sugars, on the expression of the NLRP3 inflammasome components was tested. As shown in Figure 4A, arginine and sugars (i.e., fructose, glucose, and maltose) did not induce the expressions of either pro-IL-1β or NLRP3 proteins. In addition, amino sugars (i.e., AFG and FA) did not stimulate the expression of pro-IL-1β (Figure 4B). However, maltol, the other amino sugar in the RGE, upregulated both pro-IL-1β and NLRP3 proteins in a dose-dependent manner, as seen in Figure 4C. Taken together, maltol, one of the ingredients of ASEF and RGE, induces the priming step of NLRP3 inflammasome activation.

### 2.5. ASEF Stimulated the Activation of the Inflammasome by Inducing the Priming Step of Inflammasome Activation

**Figure 5:**
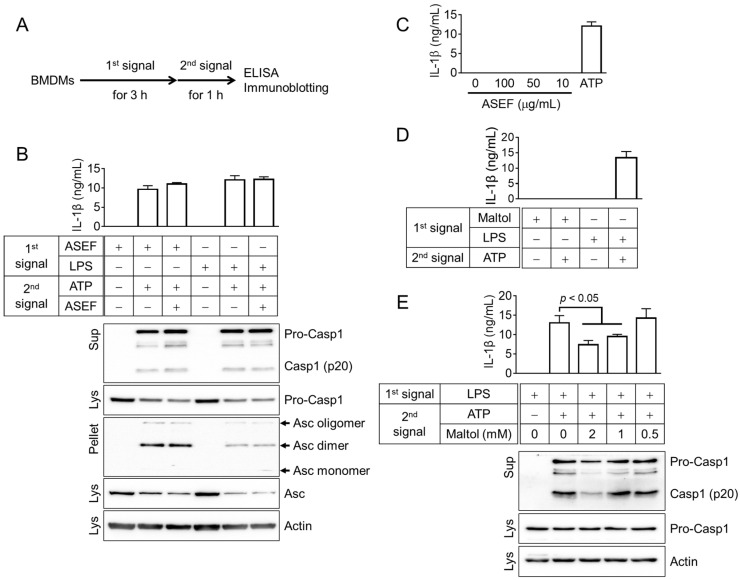
Effects of the ASEF and maltol on the priming (1st signal) or activation (2nd signal) steps of NLRP3 inflammasome activation. (**A**): Schematic diagram indicating the experimental mode for inflammasome activation. The BMDMs were treated with the ASEF (100 µg/mL), maltol (2 mM), or LPS (10 ng/mL) as the first signal for 3 h, after which the cells were replaced by media containing ATP (5 mM), the second signal, with/without ASEF or maltol for 1 h. (**B**): BMDMs were primed with ASEF or LPS and then treated with ATP to activate the NLRP3 inflammasome. IL-1β secretion was measured by ELISA and the secretion of caspase-1 (Casp1) and the formation of ASC pyroptosomes were analyzed by immunoblotting. (**C**): LPS-primed BMDMs were exposed to increasing concentrations of ASEF as indicated or ATP for 1h, and the release of IL-1β was measured using ELISA. (**D**): The BMDMs were primed with maltol or LPS and then treated with ATP to secrete IL-1β, which was measured by ELISA. (**E**): LPS-primed BMDMs were treated with maltol as indicated or ATP for 1 h, and the release of IL-1β and caspase-1 was measured using ELISA or immunoblotting. The bar graph presents the mean ± SD. All data shown are representative of at least three independent experiments.

The priming step needs to occur first in the process of cytokine maturation and pyroptosis [11]. Hence, it was evaluated whether the ASEF-primed BMDM led to inflammasome activation and whether the ASEF altered the activation step (Figure 5A). Macrophages were treated with the ASEF or LPS as a first signal for the priming step, followed by the inflammasome activation triggered by ATP. The results (Figure 5B) showed that the ASEF-primed BMDM successfully induced the secretion of IL-1β and caspase-1 (p20), as well as the formation of the ASC pyroptosome. Interestingly, the ASEF-primed BMDM enhanced the formation of ASC pyroptosome compared to the LPS-primed BMDM. The treatment of BMDMs with ASEF and ATP during the activation step did not alter the indexes of the inflammasome activation. In addition, the effect of the ASEF alone on the activation step was confirmed. The LPS-primed BMDM did not release the IL-1β in response to the ASEF alone during the activation step, while ATP did (Figure 5C). The effect of maltol on the priming and activation step was further tested. Maltol-primed BMDM did not secrete IL-1β, while the LPS-primed BMDM did (Figure 5D). Furthermore, maltol inhibited the secretion of IL-1β and caspase-1 (p20) from the LPS-primed BMDM in response to ATP during the activation step (Figure 5E and Supplementary Figure S1C). This implies that maltol induces the priming step but cannot be considered as a representative ingredient for the ASEF’s priming step enhancement due to its strong inhibition of the activation step of the NLRP3 inflammasome. Overall, it can be inferred that certain ingredients of the ASEF, such as the TLR ligands, induce the priming step of the inflammasome activation. 

## 3. Discussion

In general, it is well established that the ginsenosides of RGE inhibit the expression and inflammasome-mediated maturation of inflammatory cytokines. However, research on cytokine secretion related to the other components of RGE, such as amino sugars, has been relatively limited. In this study, it was demonstrated that the ASEF not only increased cytokine expression in macrophages but also upregulated the expression of genes associated with inflammasome components, such as NLRP3 and pro-IL-1β. Successful inflammasome activation requires two distinct steps: priming and activation. During the priming step, the gene expression of the inflammasome components is upregulated, followed by the activation of caspase-1 through protein polymerization during the activation step. The effect of the ASEF in increasing gene expression is similar to that of the TLR ligands, which induce the priming step. Through mechanistic studies, it was found that the ASEF mainly induces the priming step of the inflammasome through the TLR4 signaling pathway, with a low level of involvement of the TLR1 signaling pathway. Macrophages primed with the ASEF induced complete NLRP3 inflammasome activation, similar to cells primed with LPS. Furthermore, no inhibitory effect on priming and activation of the NLRP3 inflammasome was observed due to the ginsenosides contained in the ASEF. In this study, maltol was believed to be a component that promoted the priming step, but other ingredients of ASEF might play a role in inducing the priming step due to the distinct characteristics of maltol compared to the ASEF. Taken together, the increase in the cytokine expression and inflammasome priming induced by the ASEF may contribute to the immunomodulatory effects of ginseng.

Among the several pharmacological properties that benefit health, the effects of ginseng, including Korean Red Ginseng, on inflammation have been extensively studied. Notably, ginsenosides have been in focus as anti-inflammatory molecules. Ginsenosides modulate several inflammatory signals and pathways, including glucocorticoid receptors, NF-κB, mitogen-activated protein kinase, and antioxidants, resulting in a reduction in the production of pro-inflammatory cytokines [12,13]. Also, the role of ginseng on inflammasomes has been extensively studied. Ginseng has been shown to inhibit the activation of NLRP3, AIM2, and non-canonical (caspase-4/5 in human and caspase-11 in mice) in inflammasomes, but not the NLR family CARD domain-containing protein 4 (NLRC4) inflammasome [14,15]. Several ginsenosides (e.g., Rg1, Rg3, Rh3, Rd, and compound K) have been shown to attenuate the activation of NLRP3 and AIM2 inflammasomes [3,4]. In addition to the anti-inflammatory effects, there are several reports of the immune-boosting effects of ginseng [5]. Ginseng has been reported to increase the generation of inflammatory mediators such as nitric oxide (NO) by upregulating inducible nitric oxide synthase in macrophages, thereby enhancing phagocytic activity [16]. Notably, polysaccharides extracted from ginseng have been shown to increase IL-1β and TNFα secretion and promote NO production in macrophages and mice models, resulting in increased anti-pathogenic activity [16,17]. In a clinical study with human subjects, the intake of ginseng extract was found to increase phagocytic activity [6]. Collectively, while ginsenosides exhibit anti-inflammatory effects, non-ginsenosides exhibit immune-boosting effects. Notably, as revealed in this study, the promotion of inflammasome priming by the ASEF can be regarded as an immunity-boosting effect, as it prepares the host to be ready to fight against pathogens. ASEF-mediated enhancement of the priming step of inflammasomes might induce faster and more robust inflammatory responses (e.g., IL-1β secretion and pyroptosis) against invading pathogens in the host, effectively controlling infectious diseases. Furthermore, the presence of ASEF in RGE could serve as a marker due to its role in leading the immune-boosting effect, while ginsenosides could be considered markers of immunomodulation.

The inflammasome substrate, pro-IL-1β, is synthesized as an inactive precursor and must be cleaved or processed into a mature form (17 kDa) that interacts with the IL-1 receptors [18]. Two signals are needed to induce the two steps of inflammasome activation, namely priming, and activation for the production of pro-IL-1β and the release of mature IL-1β, respectively [10,19]. Inflammasome activation occurs in two phases due to the need for strict control over the powerful inflammatory response and cellular damage caused by NLRP3 inflammasome activation [11]. The priming step is a key component of the control measures needed to ensure a timely and appropriate inflammatory response [11]. Pro-IL-1β synthesis is generally induced by many stimuli, including TLR ligands and cytokines such as TNF-alpha [18]. However, in general, LPS stimulation through TLR4 induces pro-IL-1β production [10]. Transcriptional increases in inflammasome substrates (i.e., pro-IL-1β) occur during priming. Although pro-IL-18 is constitutively expressed, it is also increased by TLR signals [11]. Therefore, increasing the concentration of substrates through priming is essential for inflammasome activation [11]. The inflammasome sensor protein, NLRP3, itself, is also increased through NF-κB signaling in the priming step, and high levels of NLRP3 expression are essential for NLRP3 inflammasome activation [11]. To summarize, the increased expression of substrates and the sensor protein NLRP3 during priming appears to be correlated with inflammasome activation. However, the post-transcriptional modifications (e.g., ubiquitination and phosphorylation) of inflammasome components during the priming step may be additional signals as NLRP3 activation can occur even when protein synthesis is inhibited during the priming step [11,20]. Although this study did not investigate the probable post-transcriptional modifications caused by the ASEF, the study confirmed that the ASEF clearly increases transcriptional priming and induces inflammasome activation.

## 4. Conclusions

In this study, the role of the ASEF, an amino sugar-enriched fraction that has the same ginsenoside content as RGE, in the priming step of NLRP3 inflammasomes was investigated. Ginsenosides, the representative anti-inflammatory components of RGE, not only inhibit the expression of cytokines but also block the priming step. ASEF enhances the priming step despite containing a concentration of ginsenosides similar to that of RGE. Thus, it implies that the non-ginsenoside ingredients of RGE, such as amino sugars, play a more crucial role than ginsenosides in the priming step. Among the major amino sugars in ASEF, maltol stimulated the priming step. However, since maltol inhibited the activation step of inflammasome signaling, it is not suitable as a candidate of RGE’s immune-boosting substance. Although it could not be elucidated in this study, it is presumed that there is another compound or combination of compounds in ASEF that stimulates the priming step. The promotion of the priming step can expedite cytokine maturation and stimulate pyroptosis in response to pathogen invasion, leading to a faster and more robust immune response [4,21]. Thus, this study suggests a mechanism for the immune-boosting effect of RGE.

## 5. Materials and Methods

Unless otherwise indicated, all materials were purchased from Daejung Chemicals & Metals Co., Ltd. (Siheung-si, Republic of Korea), Welgene (Gyeongsan-si, Republic of Korea), or Life Sciences (Pocheon-si, Republic of Korea).

### 5.1. Preparation of ASEF, AFG, and FA

The ASEF was obtained from the Korea Ginseng Corporation (KGC, Daejeon, Republic of Korea). To prepare the ASEF, the powdered RGE (KGC, Daejeon, Republic of Korea) was dissolved in absolute ethanol (EtOH), and then the EtOH was removed. The residue was re-dissolved in water, and the eluted water fraction was evaporated to dryness in vacuo. Through this process, the concentration of ginsenosides did not change significantly, but the concentration of amino sugars, namely AFG and maltol, was increased to about three-fold in the ASEF when compared to that in RGE (Supplementary Table S1) [22,23]. 

### 5.2. Analysis of Contents of Contents of ASEF

**Figure 6:**
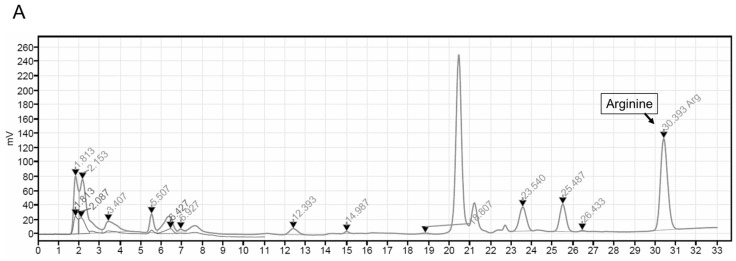

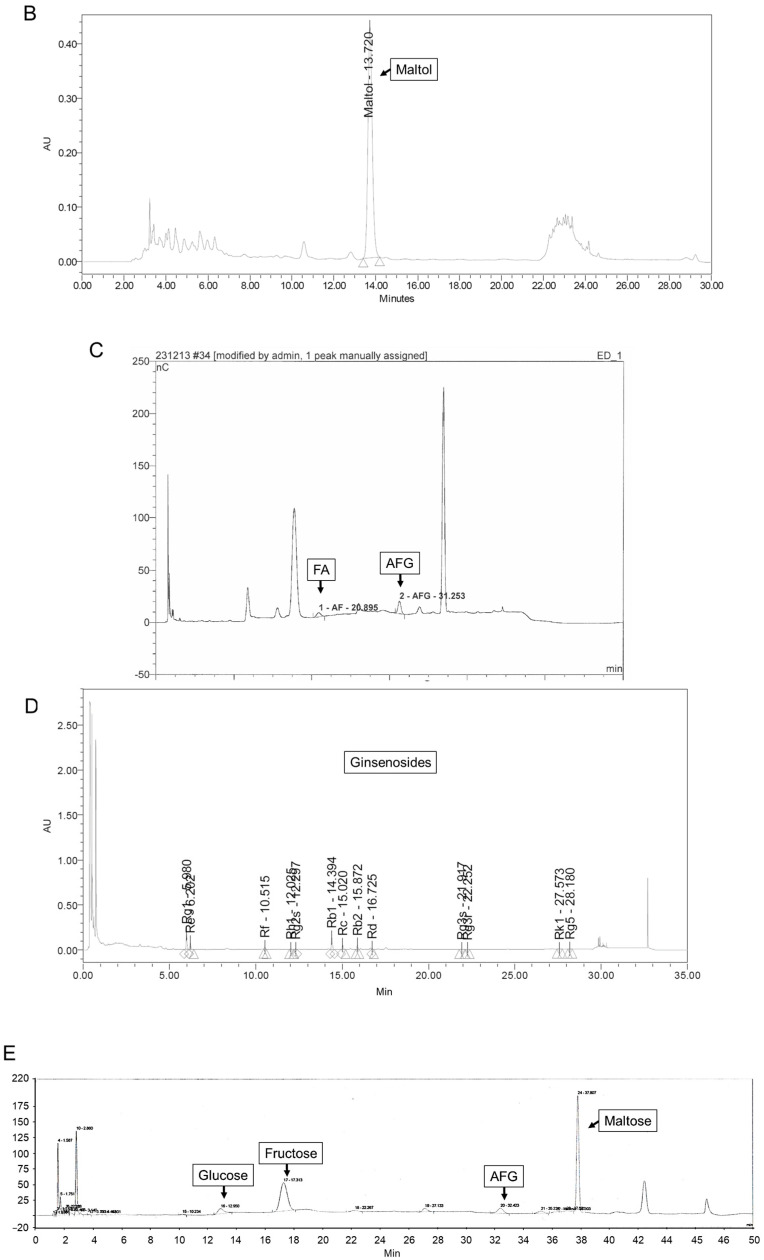
Representative chromatograms of arginine (**A**), maltol (**B**), FA and AFG (**C**), ginsenosides (**D**), and sugars (**E**) in ASEF. The analysis of ASEF was conducted by the analysis team at the Korea Ginseng Research Institute (Korea Ginseng Corporation, Daejeon, Republic of Korea). The chromatograms were analyzed, and the constituents were determined by comparing them with standard solutions in accordance with previous studies [7,22,23,24]. Triangles and diamond symbols indicate the starting and ending points of each peak.

The ASEF was analyzed for arginine, sugars, amino sugars, and ginsenosides by chromatographic analysis, as shown in Figure 6 and Table 1, in accordance with previous studies [7,22,23,24]. Briefly, the standard materials were purchased from Chromadex (Irvine, CA, USA) for ginsenosides, from Ambo Institute (Seoul, Republic of Korea) for ginsenosides and amino sugars, and from Sigma-Aldrich (Supelco Inc., Burlington, MA, USA) for arginine, sugar, and amino sugars. In total, 0.3 g of ASEF dissolved in 20 mL of methanol (70%) for ginsenosides and 0.1 to 0.5 g of ASEF dissolved in 10 mL of distilled water for arginine, sugars, and amino sugars were mixed using an ultrasonicator (SD-200H, Sungdong Ultrasonics, Seoul, Republic of Korea). For the analysis of amino acids, 1 mL of the ASEF solution in distilled water was added to 10 mL of 0.02 N HCl and mixed. After centrifugation, the supernatant was filtered through a PVDF membrane filter (0.22 µm, φ47 mm, Nylon, Whatman, Maidstone, Kent, UK). The filtrates were subjected to the following chromatographic analyses: Ginsenosides were analyzed using a UPLC/PDA system (Column: ACQUITY UPLC BEH Phenyl [2.1 × 100 mm, 1.7 µm], Detector: UV 203 nm, Acquity UPLC System, Waters, Milford, MA, USA); Maltol was analyzed using an HPLC/UVD system (Column: Discovery C18 [4.6 × 250 mm, 5 µm], Detector: UV 275 nm, Alliance 2695 HPLC system, Waters); Sugars, FA, and AFG were analyzed using a Bio-LC/PAD system (CarboPac PA-10 column [4 × 250 mm], Au working electrode and Ag/AgCl reference electrode, Dionex, Sunnyvale, CA, USA); and Arginine was analyzed using an LA8080 amino acid analyzer (Hitachi, Tokyo, Japan). Arg, arginine; AFG, arginine–fructose–glucose; FA, fructose–arginine. AFG and FA were produced using the methods described in Supplementary Materials [25].

### 5.3. Cell Culturing

To generate BMDMs, the bone marrow cells were isolated from the femur and tibia of mice (C57BL/6, Nara Biotech, Seoul, Republic of Korea) and incubated in differentiation media for 7 d. The differentiation media contained fetal bovine serum (FBS, 10%), antibiotics, and L929 cell-conditioned media (50%), which included the macrophage colony-stimulating factor. The above steps to generate BMDMs were based on previously published studies [8,9,23]. All animal experiments were carried out in accordance with the Guide for the Care and Use of Laboratory Animals, National Institutes of Health, USA, and approved by the Institutional Animal Care and Use Committee of Kangwon National University (IACUC; approval no. KW-230328-2).

### 5.4. Cell Treatment

For mRNA and protein expression assays, BMDMs were seeded in culturing plates, and then the cells were treated with the ASEF (100 µg/mL), heat-killed *Listeria monocytogenes* (HKLM, 1%), or LPS (10 ng/mL; Sigma-Aldrich Co., St. Louis, MO, USA) with/without Anti-mTLR2-IgG (100 ng/mL; mabg-mtlr2, InvivoGen, San Diego, CA, USA), CU-CPT22 (5 mM; 4884, Tocris Bioscience, Minneapolis, MN, USA), TAK-242 (5 µM; CLI-095, tlrl-cli95, InvivoGen), or polymyxin B (100 µg/mL; tlrl-pmb, InvivoGen) for 3 h. The BMDMs were treated with 1 mg/mL of arginine, fructose, glucose, maltose, AFG, and FA for 3 h.

For the assay of inflammasome activation, BMDMs were primed with the ASEF (100 µg/mL) or LPS (1 µg/mL) for 3 h and treated with adenosine triphosphate (ATP, 5 mM; InvivoGen) with/without ASEF (100 µg/mL) or maltol for 1 h. The cellular supernatant (Sup) was harvested in a tube, and the cells were lysed with a lysis buffer containing a proteinase inhibitor cocktail. The lysate (Lys) was collected after centrifugation, and the remaining cell pellets were treated with suberic acid bis (2 mM, Sigma-Aldrich Co.) for 1 h. The cross-linked pellets (Pellet) were collected by centrifugation and then re-suspended in a loading dye buffer [26]. 

### 5.5. Reverse Transcription Polymerase Chain Reaction (RT-PCR) and Quantitative Real-Time PCR (qPCR)

The total RNA was prepared with NucleoZOL (Macherey-Nagel Co., KG, Düren, Germany) and synthesized to complementary DNA (cDNA) by a synthesis kit (Enzynomics Inc., Daejeon, Republic of Korea). For RT-PCR, the transcripts were amplified using nTaq polymerase (Enzynomics) and a cycler (Thermo Fisher Scientific Inc. Grand Island, NY, USA). The band of PCR products was separated by agarose gel electrophoresis and visualized by staining with ethidium bromide. For qPCR, the transcripts were quantified using a PCR system (Eco Real-Time PCR, Illumina, San Diego, CA, USA) and an enzyme mixture containing SYBR Green (Enzynomics). The quantitation was normalized with a housekeeping gene (glyceraldehyde 3-phosphate dehydrogenase [GAPDH]). The gene-specific primers are provided in the Supplementary Materials.

### 5.6. Western Blot Analysis

Samples of the cellular supernatant, lysate, or pellets were separated using a sodium dodecyl sulfate polyacrylamide gel electrophoresis (SDS-PAGE) using a gel running system (Bio-Rad Laboratories, Hercules, CA, USA). The gels were further transferred onto a polyvinylidene difluoride membrane (PVDF; GE Healthcare, Chicago, IL, USA). The membranes were soaked in skim milk (5%) and then probed with an anti-mouse IL-1β antibody (AF-401-NA, R&D Systems, Minneapolis, MN, USA), anti-NLRP3 antibody (AG-20B-0014-C100, AdipoGen Life Sciences, San Diego, CA, USA), anti-caspase-1 antibody (AG-20B-0042-C100, AdipoGen), or anti-actin antibody (sc-1615, Santa Cruz Biotechnology, Dallas, TX, USA) overnight at 4 °C. The membrane was further probed with secondary antibodies conjugated with horseradish peroxidase (HRP; sc-2020, sc-2005 or sc-2004, Santa Cruz Biotechnology) for 2 h and then analyzed under a chemiluminescence solution (AbFrontier, Seoul, Republic of Korea) and a chemiluminescence imaging system (EZ-Capture II, ATTO Technology, Tokyo, Japan). 

### 5.7. Statistical Analysis

Statistical software (GraphPad Prism 6, San Diego, CA, CA) was used to perform analyses using the Mann–Whitney test or one-way ANOVA. The p-value is shown in the figures.

## Figures and Tables

**Table 1:** Contents (mg/g) of ASEF.

Arg	Amino Sugars			Ginsenosides									
	AFG	FA	Maltol	Rg1	Re	Rf	Rh1	Rg2s	Rb1	Rc	Rd	Rg3s	Rg3r
9.68	95.41	14.46	3.48	4.63	2.5	1.13	0.22	1.8	5.43	1.9	0.36	0.11	0.05

ASEF: amino sugar-enriched fraction; Arg, arginine; AFG, arginine–fructose–glucose; FA, fructose–arginine.

## Data Availability

Data are contained within the article and Supplementary Materials.

## Supplementary Materials

The following supporting information can be downloaded at: https://www.mdpi.com/article/10.3390/molecules29071455/s1, Figure S1: Effects of the ASEF on inflammasome priming, Table S1: Comparison of the saponin and non-saponin contents between RGE and its sub-fraction [23], Materials and Methods: Preparation of AFG and FA, and the gene-specific primers.
