# Supplementary material for: Amino Sugar-Enriched Fraction of Korean Red Ginseng Extract Induces the Priming Step of NLRP3 Inflammasome
Source: Molecules. 2024 Mar 24;29(7):1455. doi: 10.3390/molecules29071455 (PMC11013037; doi:10.3390/molecules29071455)
Supplement: Supplementary file 1 [file molecules-29-01455-s001.zip › molecules-2905954-supplementary.pdf]

Supplementary Information

Amino sugar-enriched fraction of Korean Red Ginseng extract induces the priming step of NLRP3 inflammasome

Huijeong Ahn[1] and Geun-Shik Lee[1]*

[1]College of Veterinary Medicine and Institute of Veterinary Science, Kangwon National University, Chuncheon, Republic of Korea

*Correspondence: Geun-Shik Lee, D. V. M., Ph. D.

Laboratory of Inflammatory Diseases, Department of Physiology, College of Veterinary Medicine, Kangwon National University, Chuncheon, Gangwon, 24341, Republic of Korea.

e-mail: leegeun@kangwon.ac.kr, Tel: +82-33-250-8683, Fax: +82-33-244-2367

Supplementary Figure S1

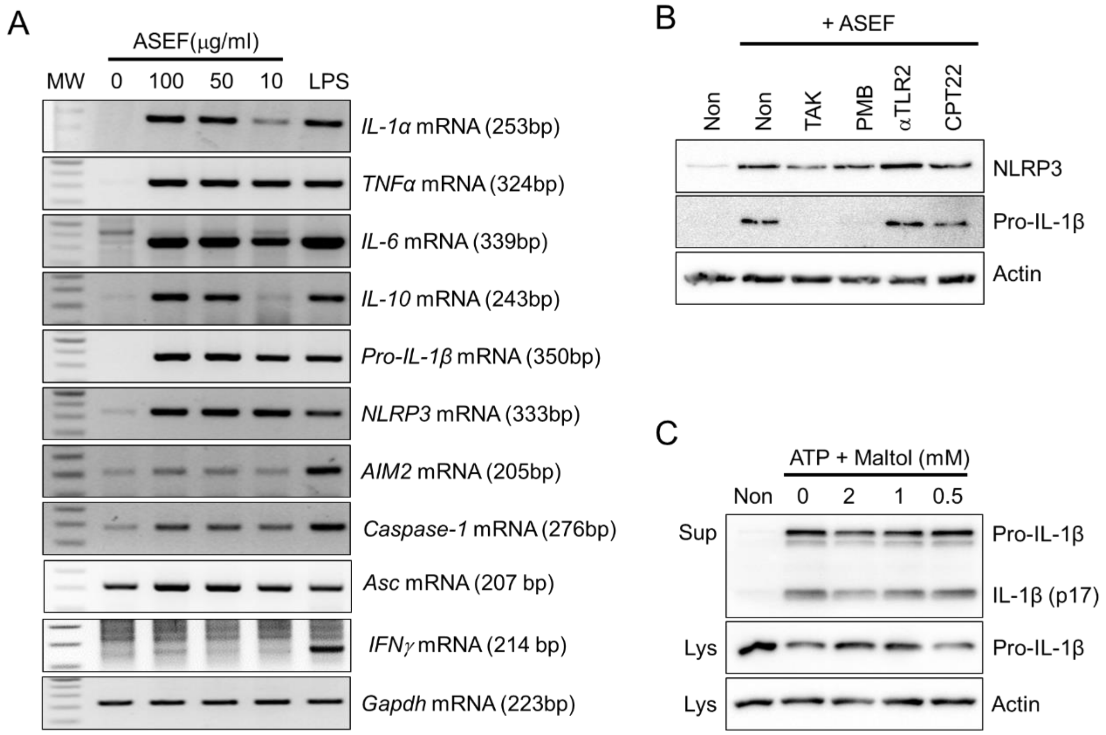

Supplementary Fig. S1. Effects of the ASEF on inflammasome priming

A, BMDM were treated with the indicated concentrations of the ASEF or LPS (10 ng/mL) for 3h. The gene expression level was analyzed by RT-PCR. **B**, BMDM were treated with ASEF (100 μg/mL) in the presence of TLR inhibitors (CU-CPT22 [CPT22] for TLR1/2, αTLR2 for TLR2, TAK for TLR4, and PMB for LPS) for 3h. NLRP3 and pro-IL-1β proteins were analyzed by immunoblotting. **C**, LPS-primed BMDM were treated with maltol as indicated or ATP for 1h, and the release of IL-1β was measured using immunoblotting.

Supplementary Table S1. Comparison of the saponin and non-saponin contents in RGE and its sub-fractions [Ref. S4]

Components (mg/g)		ASEF	RGE	SF	NS
Ginsenosides	Rg1	4.63	1.06	13.33	0.35
	Re	2.5	1.21	15.68	0.40
	Rf	1.13	1.04	13.39	0.35
	Rh1	0.22	0.96	11.54	0.32
	Rg2s	1.8	1.43	15.75	0.48
	Rb1	5.43	5.19	64.20	1.73
	Rc	1.9	2.02	25.98	0.67
	Rd	0.36	0.67	8.29	0.22
	Rg3s	0.11	2.10	21.69	0.70
	Rg3r	0.05	0.96	10.60	0.32
	Sum	18.13	16.5	197.43	5.5
AFG		95.41	28.18	6.75	39.78

ASEF, amino-sugar enriched fraction; RGE, Korean Red Ginseng extract; SF, saponin fraction; NS, non-saponin fraction; AFG, arginine-fructose-glucose

Ref S4. Ahn H, Han BC, Kim J, Kang SG, Kim PH, Jang KH, So SH, Lee SH, Lee GS. Nonsaponin fraction of Korean Red Ginseng attenuates cytokine production via inhibition of TLR4 expression. Journal of ginseng research 2019;43:291-9.

Supplementary Materials and Methods

Preparation of AFG and FA

Arginine and dextrose (100 mg each) for FA synthesis and arginine and maltose (100 mg each) for AFG synthesis were dissolved in glacial acetic acid (10 mL) and the solutions were incubated in a water bath at 80 °C for 1h. After completion of the Maillard reaction, the synthesized AFG and FA were dried using a vacuum evaporator and solubilized in water (up to 10 mL; 100 mg/mL).

The gene-specific primers

IL-1α (Genebank ID: NM_010554) 5’-CCG ACC TCA TTT TCT TCT GG-3’ and 5’-GTG CAC CCG ACT TTG TTC TT-3’; *tumor necrosis factor* (*TNF*) α (NM_013693) 5’-ACG GCA TGG ATC TCA AAG AC-3’ and 5’-GTG GGT GAG GAG CAC GTA GT-3’; *IL-6* (NM_031168) 5’- GTT CTC TGG GAA ATC GTG GA-3’ and 5’-GGA AAT TGG GGT AGG AAG GA-3’; *IL-10* (NM_010548) 5’-TCA TTT CCG ATA AGG CTT GG-3’ and 5’-TGC TAT GCT GCC TGC TCT TA-3’; *pro-IL-1β* (NM_008361) 5’-CCC AAG CAA TAC CCA AAG AA-3’ and 5’-GCT TGT GCT CTG CTT GTG AG-3’; *NLRP3* (NM_145827) 5’-CAG GCG AGA CCT CTG GGA AA-3’ and 5’-CCC AGC AAA CCC ATC CAC TC-3’; *caspase-1* (NM_009807) 5′-CTG GGA CCC TCA AGT TTT GC-3′ and 5′-GGC AGG CAG CAA ATT CTT TC-3′; *Asc* (NM_023258) 5′-CAG AAG TGG ACG GAG TGC TG-3′ and 5′-AGC TCT GCT CCA GGT CCA TC-3′; *AIM2* (NM_001013779) 5′-AGT GGC CAC GGA GAC AGA TT-3′ and 5′-GGG AGT TTC CCT GGC TCT CT-3′; *GAPDH* (NM_001289726) 5’-AAC TTT GGC ATT GTG GAA GG-3’ and 5’-ACA CAT TGG GGG TAG GAA CA-3’.
